# Genetic issues in ICP

**DOI:** 10.1177/1753495X241263441

**Published:** 2024-07-26

**Authors:** Julia Zöllner, Catherine Williamson, Peter H Dixon

**Affiliations:** 1 4919 University College London, London, UK; 2 Imperial College London, London, UK

**Keywords:** Intrahepatic cholestasis of pregnancy, genetics, genomics, pregnancy complications

## Abstract

Intrahepatic cholestasis of pregnancy (ICP) is the commonest gestational liver disorder with variable global incidence. Genetic susceptibility, combined with hormonal and environmental influences, contributes to ICP aetiology. Adverse pregnancy outcomes linked to elevated serum bile acids highlight the importance of comprehensive risk assessment. *ABCB4* and *ABCB11* gene variants play a significant role in about 20% of severe ICP cases. Several other genes including *ATP8B1*, *NR1H4*, *ABCC2*, *TJP2*, *SERPINA1*, *GCKR* and *HNF4A* have also been implicated with ICP. Additionally*, ABCB4* variants elevate the risk of drug-induced intrahepatic cholestasis, gallstone disease, gallbladder and bile duct carcinoma, liver cirrhosis and abnormal liver function tests. Genetic variations, both rare and common, intricately contribute to ICP susceptibility. Leveraging genetic insights holds promise for personalised management and intervention strategies. Further research is needed to elucidate variant-specific phenotypic expressions and therapeutic implications, advancing precision medicine in ICP management.

## Key findings

- Genetic susceptibility, hormonal and environmental factors contribute to the development of ICP.
- Both common and rare genetic variations increase the susceptibility to ICP during pregnancy.
- Homozygous variants in *ABCB4* and *ABCB11* (bile constituent transporters) cause severe cholestasis (PFIC), while heterozygous variants increase ICP risk and are thought to be involved in >20% of ICP cases.
- Other genes including *ATP8B1*, *NR1H4*, *TJP2,* and *ABCC2* have been implicated with lesser certainty to ICP.
- Identifying susceptibility variants through testing can inform personalised management, holds promise for targeted therapies and prevention strategies.

## Introduction

Intrahepatic cholestasis of pregnancy (ICP) is the commonest gestational liver disease.^1^ The worldwide incidence varies widely (<1% to 4%) and may be explained by differences in susceptibility between ethnic groups as well as environmental factors.^2-4^ The highest incidence recorded worldwide was previously reported in the native Andean population in Chile (27.6%)^2^ although this has fallen in recent years. In the UK, the incidence is 0.62% in women of European origin, and approximately twice as common in women of Indian (1.24%) and Pakistani (1.46%) origin.^5^

ICP is characterised by pruritus presenting in the third trimester, elevated serum bile acids and abnormal tests of liver function. Adverse pregnancy outcomes, including preterm birth, fetal asphyxia, meconium-stained amniotic fluid and stillbirth, occur more commonly in pregnancies where maternal serum bile acid concentrations are ≥40 µmol.^6,7^ The recurrence risk is as high as 60–70% in subsequent pregnancies.^8^ Recent evidence demonstrates a significant increase in stillbirth risk with serum bile acid concentrations ≥100 µmol from 35 weeks’ gestation.^9^ A diagnosis of ICP is clinically significant due to its implications extending beyond the immediate pregnancy. These include an elevated risk of subsequent hepatobiliary disorders, encompassing cancer, immune-mediated conditions and cardiovascular diseases.^3^ Furthermore, there is an increased prevalence of gallstone-related illnesses and a correlation between ICP and hepatitis C.^10^

## The role of genetics

**Figure 1.**
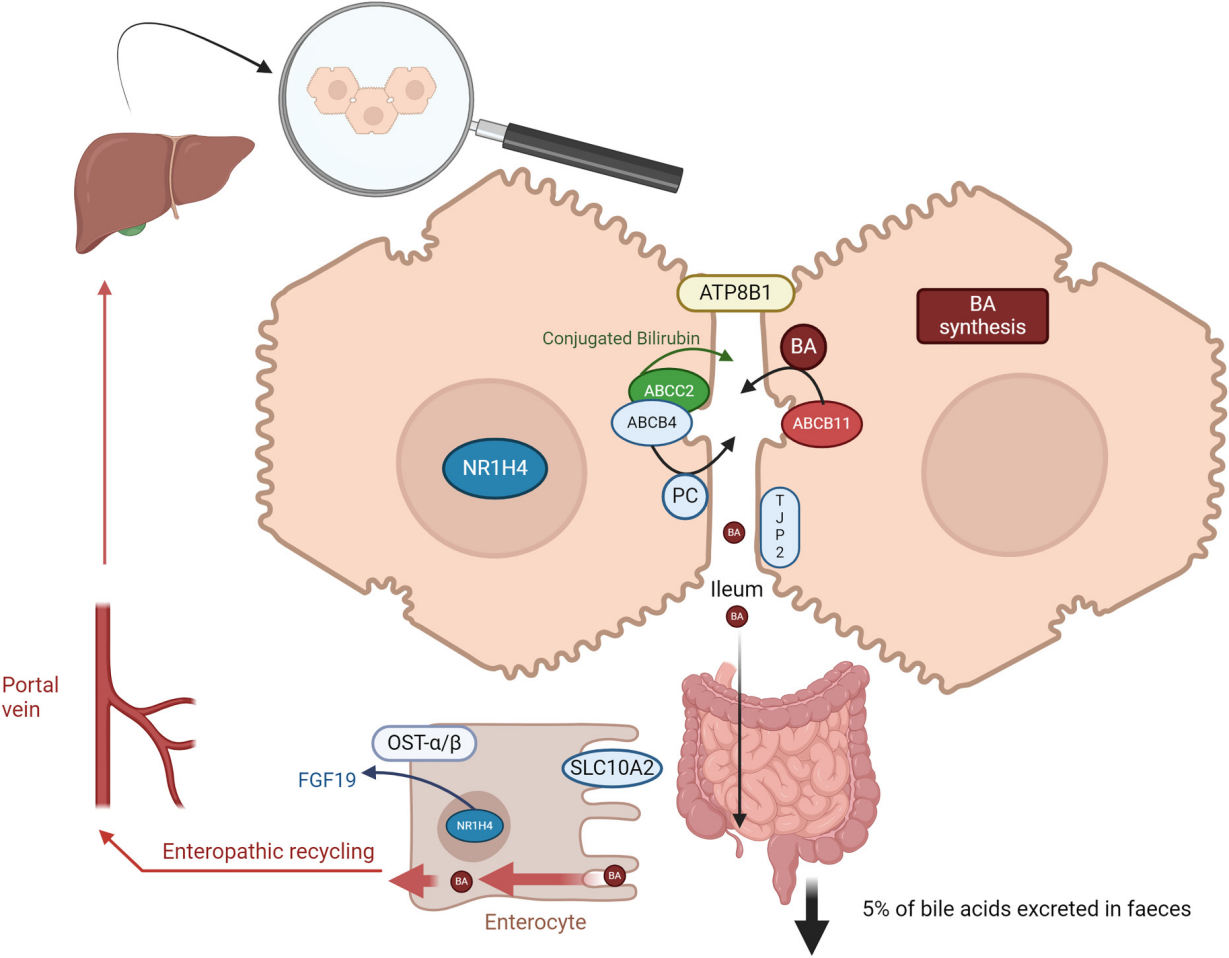
The role of canalicular transporters or their regulators implicated in the pathogenesis of ICP.

BA: bile acids; PC: phosphatidyl choline. Created with BioRender.com.

The aetiopathogenesis of ICP is not well understood but genetic susceptibility, hormonal and environmental factors are believed to combine to cause ICP. Nutritional deficiencies in selenium and vitamin D, prevalent in specific regions and seasons, have been associated with increased ICP risk.^11^ Additionally, the hormonal surge during pregnancy, particularly high oestrogen and progesterone levels, can trigger cholestasis in genetically predisposed individuals.^11^ The genetic basis of ICP is supported by evidence of familial clustering,^12,13^ increased risk in first degree relatives^14^ and population specific risk differences.^15^ Thus far, the majority of genetic ICP studies have explored rare variation with minor allele frequencies <1%, as they thought to have larger effect sizes and more deleterious impact on protein function. Most studies have interrogated multiple European populations but are failing on diversity. Several genes have been implicated in the aetiopathogenesis of ICP including the *ABCB4* (adenosine triphosphate-binding cassette (ATP), subfamily B, member 4) gene coding for the multidrug resistance 3 (MDR3) protein (a canalicular phospholipid translocator), and the *ABCB11* (ATP-binding cassette, subfamily B, member 11) gene coding for the bile salt export pump (BSEP) (Figure 1). Homozygous variants or compound heterozygous variants in *ABCB4* and *ABCB11* can cause a spectrum of disease from moderate cholestasis to severe progressive familial intrahepatic cholestasis (PFIC), PFIC3 and PFIC2 respectively. Variants in *ABCB4* and *ABCB11* are involved in up to 25% of patients with severe ICP.^16^ *ABCB4* variants also increase the risk of developing drug-induced intrahepatic cholestasis, gallstone disease, gallbladder and bile duct carcinoma, liver cirrhosis and abnormal liver function tests.^17^ Variants in several other canalicular transporters or their regulators are implicated in the pathogenesis of ICP, for example, *ATP8B*1 (ATPase phospholipid transporting 8B1),^18^ *NR1H4* (FXR, a principal bile acid sensor) gene^19,20^ and *TJP2* (tight junction protein 2),^21^ although supporting data are more limited. A recent study has demonstrated a possible role for *ABCC2* (ATP-binding cassette sub-family C member 2) variants, which encodes a protein that is expressed in the canalicular (apical) part of the hepatocyte and functions in biliary transport^22^ (Figure 1).

There is increasing evidence that common genetic variants (minor allele frequencies >5%) contribute to disease susceptibility, albeit to a much smaller degree than pathogenic variants.^23^ A recent study, conducted in 1138 cases and 153,642 controls implicated 11 loci in liver-enriched genes and liver-specific cis-regulatory elements as contributing mechanisms to ICP susceptibility. For example, they found that a single missense coding variants in *SERPINA1* (associated with cystic fibrosis liver disease)*, GCKR* and *HNF4A* (both associated with hepatic metabolic homeostasis) had strong evidence for association, amongst other signals.^23^ Therefore, both common and rare variants contribute to the genetic risk of ICP and can explain variability in disease.

## ABCB4

The ABCB4 protein plays a crucial role in the transportation of phospholipids across cell membranes, specifically in the liver. Phospholipids in the bile function to solubilise bile acids, preventing them from causing harm. In addition, the phospholipid coat helps maintain the integrity of the bile canaliculi. Multiple heterozygous variants in the *ABCB4* gene (chromosome 7, 7q21) have been reported in women with ICP^16,21,22,24-40^ (Table 1). There are reports that some variants or a combination of variants is associated with a more severe phenotype but no functional data is available to confirm this.^34^ Additionally, common variants of the *ABCB4* gene have been identified as genetic risk factors for the severe form of ICP in Sweden.^22,39^ The role of *ABCB4* in ICP is further supported by its association with drug-induced cholestasis, where genetic variants of *ABCB4* and *ABCB11* have been implicated^41^ including hormonal contraception.^42^

**Table 1.** A summary of important rare and common genetic studies in ICP with a focus on *ABCB4*, *ABCB11* and *ATP8B1 genes*.

Author	Study type	Number of participants	Gene	Interpretation
Jacquemin 1999^24^	Case study of a large consanguineous family	6	*ABCB4*	Identical heterozygous *ABCB4* variant in all cases (NC_000007.14(NM_000443.4):c.1712del; (NP_000434.1):p.(Val571Aspfs*16))
Dixon 2000^25^	Case study	8	*ABCB4*	Heterozygous variant in 1 participant (NC_000007.14(NM_000443.4):c.1637C > A; (NP_000434.1):p.(Ala546*Asp*))
Rosmorduc 2001^26^	Case study	6	*ABCB4*	Heterozygous variant in 1 case (NC_000007.14(NM_000443.4):c.1327_1328insT; (NP_000434.1):p.(Gln443Leufs*5)), homozygous variant in 2 cases (NC_000007.14(NM_000443.4):c.959C > T; (NP_000434.1):p.(Ser320Phe))
Lucena 2003^27^	Case study	1	*ABCB4*	Heterozygous variant (NC_000007.14(NM_000443.4):c.1604G > C; (NP_000434.1):p.(Gly535Ala))
Müllenbach 2003^28^	Case study & Case-control	14	*ABCB4*	Heterozygous variant (NC_000007.14(NM_000443.4):c.449G > A; (NP_000434.1):p.(Arg150Lys))
Eloranta 2003^43^	Case-control study	57 cases & 115 controls	*ABCB11*	SNPs rs853782 (NC_000002.12(NM_003742.2):c.1331T > C; (NP_003733.2):p.(Val444Ala)) and rs473351 (NC_000007.14(NM_000443.4):c.1712T > A; (NP_000434.1):p.(Val571Glu)), (*P* = 0.04 and *P* = 0.02, respectively)
Pauli-Magnus 2004^29^	Case-control study	21 cases & 40 controls	*ABCB4* & *ABCB11*	3 heterozygous variants (*ABCB11*, NC_000002.12(NM_003742.2):c.1772A > G; (NP_003733.2):p.(Asn591Ser); *ABCB4*, NC_000007.14(NM_000443.4):c.959C > T; (NP_000434.1):p.(Ser320Phe) and NC_000007.14(NM_000443.4):c.2285G > A; (NP_000434.1):p.(Gly762Glu))
Floreani 2006^30^	Case-control study	80 cases & 80 controls	*ABCB4*	3 heterozygous variants (NC_000007.14(NM_000443.4):c.1584G > C; (NP_000434.1):p.(Glu528Asp), NC_000007.14(NM_000443.4):c.1646G > A; (NP_000434.1):p.(Arg549His), NC_000007.14(NM_000443.4):c.1606G > C; (NP_000434.1):p.(Gly536Arg))
Wasmuth 2007^39^	Case-control study	52 cases & 52 controls	*ABCB4* & *ABCB11*	*ABCB4* haplotypes differed between the two groups (*P* = 0.019), but not in *ABCB11*
Pasmant 2012^31^	Case study	59 ICP/CIC cases	*ABCB4*	*ABCB4* point or short insertion/deletion variants found in 27%
Gotthardt 2008^32^	Case study of a family with distal consanguinity	9	*ABCB4*	6 cases demonstrated heterozygous variant (NC_000007.14(NM_000443.4):c.2362C > T; (NP_000434.1):p.(Arg788Trp))
Dixon 2009^33^ *	Case-control study	491 cases & 261 controls	*ABCB11*	1.4% had *ABCB11* variant. Common V444A association found
Anzivino 2013^34^	Case-control study	33 cases & 100 controls	*ABCB4* & *ABCB11*	33% had a variant (5 *ABCB4*, 6 *ABCB11*), none in controls
Dixon 2014^35^	Case-control study	563 cases & 642 controls	*ABCB4, ABCB11* &	Evidence of common polymorphisms (strongest association in rs2109505 NC_000007.14(NM_000443.4):c.711A > T; (NP_000434.1):p.(=) in *ABCB4* and with rs7577650 NC_000002.12:g.169034700G > A in *ABCB11*)
Dixon 2017^21^	Cohort of ICP	147	*ABCB11, ABCB4,* & *ATP8B1*	14% had a variant (12 *ABCB4*, 4 *ABCB11*, 5 *ATP8B1*)
Yeap 2018^40^	Case study	5	*ABCB4* & *ABCB11*	Heterozygous and homozygous variants in *ABCB4* and *ABCB11*
Truro 2020^16^	Whole genome sequencing	310 severe early onset ICP cases	*ABCB4* & *ABCB11*	20% of cases presented with variants
Aydin 2020^18^	Case study	25	*ABCB4, ACBC11* & *ATP8B1*	Heterozygous variants observed *ATP8B1* (*n* = 2), *ABCB11* (*n* = 1) and *ABCB4* (*n* = 7)
Liu 2021^22^	Whole exome sequencing	151 cases	44 *ABC* family transporter genes	*ABCB4* (NC_000007.14(NM_000443.4):c.2123G > A; (NP_000434.1):p.(Trp708*), NC_000007.14(NM_000443.4):c.1580G > A; (NP_000434.1):p.(Gly527Glu) and NC_000007.14(NM_000443.4):c.1156A > G; (NP_000434.1):p.(Lys386Glu))*, ABCB11* (NC_000002.12(NM_003742.2):c.3580C > T; (NP_003733.2):p.(Gln1194*), NC_000002.12(NM_003742.2):c.1814A > C ; (NP_003733.2):p.(Gln605Pro) and NC_000002.12(NM_003742.2):c.1765C > A; (NP_003733.2):p.(Leu589Met))*, ABCC2* (NM_000392.4:r.(4025c > a); (NP_000383.1):p.(Ser1342Tyr))
Müllenbach 2005^36^	Case-control study	182 cases & 120 controls	*ATP8B1*	Two heterozygous variants (NM_005603.4:r.(208g > a) ; (NP_005594.1):p.(Asp70Asn) and NM_005603.4:r.(2599c > u); (NP_005594.1):p.(Arg867Cys)) in 3 cases
Painter 2005^37^	Case-control study	176 cases & 100 controls	*ATP8B1*	3% of cases had a variant versus none in controls
Zöllner 2023^38^	Whole exome sequencing	5000 volunteers – gene candidate approach	*ABCB4, ABCB11* & *ATP8B1*	Novel variants identified in a British Bangladeshi and Pakistani cohort associated with ICP and cholestatic liver disease
Dixon 2022^23^*	GWAS meta-analysis	1138 cases and 153,642 controls	11 loci identified	*GCKR, ABCG5/8, ABCB11, SCARB2, ABCB1/4, CYP7A1, SERPINA1, ENPP7, TMEM147, SULT2A1, HNF4A*

AF: allele frequency; CIC: contraceptive-induced cholestasis; GWAS: genome wide association studies; ICP: intrahepatic cholestasis of pregnancy; *common variation studies.

To date, there are 102 reported variants associated with ICP in the Clinvar database (https://www.ncbi.nlm.nih.gov/clinvar/; a freely available, public archive of human genetic variants and interpretations of their significance to disease). Of those 11 are considered pathogenic and 4 as likely pathogenic.^44^

## ABCB11

ABCB11 actively transports conjugated bile acids from hepatocytes into the bile canaliculi. This unidirectional pump mechanism operates against a concentration gradient, efficiently clearing the cytoplasm of potentially toxic bile acids and funnelling them into the digestive tract. A number of heterozygous variants in the *ABCB11 *gene (chromosome 2, 2q24) have been implicated in the development of ICP^16,18,21,22,29,33-35,38-40,43^ (see Table 1). Despite sometimes being classified as benign the *ABCB11* variant V444A (RefSeq: NP_003733.2) paints a puzzling picture. Evidence suggests it may contribute to an increased risk of ICP, progression of hepatitis C disease and even drug-induced liver injury.^29,33^ The exact mechanisms through which it exerts these effects, however, remain not understood. This apparent disconnect between classification and observed associations underscores the complexity of our understanding of genetic variants and their diverse clinical implications.^33,45^ In Clinvar, a smaller number of variants (*n* = 12) have been reported in *ABCB11,* of which four are pathogenic, and five are considered likely pathogenic.

## ATP8B1 (FIC-1)

The lack of ATP8B1 is associated with an asymmetry of phospholipids in the canalicular membrane, decreasing the bile secretion capacity of the liver. Although the *ATP81B* gene (chromsome 18, 18q21.31) product is not directly involved in the bile acid transport, it may affect the bile acid transporters like BSEP and their functions, contributing to the disease aetiopathogenesis. Although there is less evidence of the implications of the *ATP8B1* gene in the involvement of ICP there have been several studies published that have demonstrated heterozygous variants associated with ICP^18,21,36-38^ (see Table 1). In Clinvar out of 19 there is only 1 likely pathogenic and one pathogenic variant reported.

## Conclusion

Common and rare genetic variations play a complex and interdependent role in the development of ICP. While common variants increase susceptibility to the condition by two- to three-fold, rare variants are believed to cause ICP. Both types of variations can interact with environmental and hormonal factors. There is a clear role for the use of genetic information to improve diagnosis, personalise care and implement prevention strategies. Additional research is needed to determine if specific genetic variants or combinations of variations lead to more severe phenotypes or if other factors contribute to adverse outcomes. Identifying genetic susceptibility through testing can help to inform personalised management strategies, including early intervention after pregnancy for women affected by ICP but also their relatives. Understanding the functional consequences of specific variants can also pave the way for targeted therapies in the future.
